# Supplementary material for: A Pregnancy and Childhood Epigenetics Consortium (PACE) meta-analysis highlights potential relationships between birth order and neonatal blood DNA methylation
Source: Commun Biol. 2024 Jan 9;7:66. doi: 10.1038/s42003-023-05698-x (PMC10776586; doi:10.1038/s42003-023-05698-x)
Supplement: Supplementary file 2 — Supplementary Information [file 42003_2023_5698_MOESM2_ESM.pdf]

Supplementary Note 1: Description of cohorts

LiNA cohort

Design and study population: Lifestyle and environmental factors and their Influence on Newborns Allergy risk (LiNA). Cohort profile please see [1 2 3]. The LINA study is a running prospective birth cohort study conducted by the Helmholtz Centre for Environmental Research-UFZ in Leipzig, Germany, with the aim to entangle the impact of environmental factors on the maturation of children's immune system and the development of allergic diseases, obesity, and behavioral disorders. For this study, 629 mother-child pairs were recruited from 2006 until 2008 in the city of Leipzig, Germany. Pregnant women were invited to participate, and after informed consent, maternal blood, and urine samples as well as questionnaire data were collected around the 34th week of gestation. Since birth, children as well as mothers are followed up annually by standardized questionnaires and clinical visits including blood, urine, and feces collection. In maternal and in children's blood, several immune, metabolome and gene expression analyses have been performed. Methylation analysis (450K) have been performed in cord blood samples (n=472).

Consent and ethical approval: All participants gave written informed consent. The LINA study was approved by the Institutional Review Board of the University of Leipzig and the Saxonian Board of Physicians (046-2006, 160-2008, 160b/2008, 144-10-31052010, 113-11-18042011, 206-12-02072012, 169/13-ff, 150/14-ff, EK-allg-28/14-1).

Blood collection and DNA extraction: Genomic DNA was isolated from cord blood samples using the QIAmp DNA Blood Mini Kit (Qiagen, Hilden, Germany) followed by bisulfite conversion using the EZ-96 DNA Methylation Kit (Zymo Research Corporation, Orange, USA) according to the manufacturer's recommendations. All samples subsequently subjected to DNA methylation analyses passed the initial quality control check (n = 472). A genome-wide DNA methylation screen was performed based on the Infinium HumanMethylation450 BeadChip (Illumina, San Diego, USA) array (GPL13534 platform).

Preprocessing steps: Data were normalized using the SWAN (subset-quantile within array normalization) method of the minfi R package. DNA methylation values, described as beta values (β), were recorded for each locus in each sample. The β values represent the ratio of methylated signal relative to the sum of methylated and unmethylated signal measured per CpG. Outliers in the β values were removed if they were outside of the 3* IQR range for each CpG.

Ancestry/ethnicity: All children with DNA methylation data were of European ancestry.
Cell type correction: Cell counts estimated using the Salas cord blood reference panel [4] were included as covariates.

Batch correction: Adjustment for batch effects was done by including sample plate # as a covariate.

Smoking during pregnancy: In questionnaires administered at 34th week of gestation, mothers reported whether they had smoked during pregnancy with a classification of never, occasionally, and daily. In the current analyses, we used a dichotomous variable indicating sustained smokers during pregnancy vs. non-smokers/quit when pregnancy was known.
Funding:

Funding: Core funding for the LiNA study is provided by the Helmholtz Center for Environmental Research – UFZ / Department of Environmental Immunology. The part methylation analysis in the LiNA study was supported by the German Cancer Research Centre – DKFZ.

Acknowledgements: We thank Melanie Bänsch, Anne Hain, Beate Fink and Michaela Loschinski for their excellent technical assistance and field work. Furthermore, we cordially thank the LiNA children and their families for their ongoing participation and our clinical cooperation partners.

DCHS cohort

Description of the DCHS cohort: Drakenstein Child Health Study (DCHS). The DCHS, a population-based birth cohort, has been described previously [5]. Mothers were enrolled prenatally in their second trimester and followed through pregnancy at two primary care clinics serving two distinct populations (predominantly black African ancestry or predominantly mixed ancestry). Mother-child pairs were followed from birth and infants enrolled in the DCHS were followed until at least five years of age [5]. All births occurred at a single, central facility, Paarl Hospital. Ethical approval for human subjects' research was obtained from the Human Research Ethics Committee of the Faculty of Health Sciences of University of Cape Town (HREC UCT REF 401/2009; HREC UCT REF 525/2012). Written informed consent was signed by the mothers on behalf of herself andher infant for participation in this study. The present study is based on children from the DCHS with DNA methylation data from cord blood, genotyping data, and information on psychopathology factors and covariates.

Blood collection and DNA extraction: DNA was isolated from cord blood samples that were collected at time of delivery (Morin et al. 2017). DNA methylation was assessed with the Illumina Infinium HumanMethylation450 BeadChips (n=156) and the MethylationEPIC BeadChips (n=160).

Preprocessing steps: Pre-processing and statistics were done using R 3.5.1. Raw iDat files were imported to RStudio where intensity values were converted into beta values. Background subtraction, color correction and normalization were performed using the preprocessFunnorm function [6]. After sample and probe filtering, 273 samples and 409,033 probes remained for downstream analyses. Batch effects were removed using ComBat from the R package sva [7]. Cord blood cell type composition was predicted using the most recent cord blood reference data set [4] and the IDOL algorithm and probe selection [8].

Sample Filtering: Samples were determined to be outliers if detected using two or more of the following methods, detectOutlier function from the lumi package [9] and Hannum et al. method [10] using the locFDR package [11] and both the outlyx and pfilter functions from the watermelon package [12]. However, no samples we detected in more than one method and so none were removed for this reason. Samples containing maternal blood contamination (n = 33) were removed. After the completion of pre-processing technical replicates (n = 7) and samples where reported sex didn't match sex chromosome methylation signatures (n = 3) were removed leaving a total of 273 samples remaining for downstream analysis.

Probe Filtering: This dataset contains 59 probes which detect single nucleotide polymorphisms for quality control purposes and so once observed, were removed. Probes with NAs in ≥ 1% of samples or had a detection p value ≥ 1x10-16 in ≥ 1% of samples were removed

(n = 10,868). Probes which bind to the sex chromosomes were removed due to the distribution differences observed (n = 9,896). Probes whose sequence contains a SNP either at the CpG site being measured or at the site of the single base pair extension with a minor allele frequency ≥ 1% [13 14] were removed (n = 13,598). Autosomal probes which were in silico predicted to non-specifically bind to sex chromosomes in the genome were also removed (n = 9,698) leaving a total of 409,033 probes remaining for downstream analysis [13 14].

DCHS Cohort contains two datasets in this study, measured by 450K and EPIC arrays respectively.

POSEIDON Cohort

Cohort Description: The POSEIDON (Pre-, Peri-, and Postnatal Stress: Epigenetic impact on Depression) is a longitudinal study conducted in the Rhine-Neckar Region in Germany. Mothers (n=410) were recruited in the third trimester of pregnancy (T1). Following assessments were conducted: at childbirth (T2), six months after birth (T3) and 45 months after birth (T4). The first four waves of assessment took place from 2010 to 2017. T5 was an online questionnaire assessment in 2020.

Preprocessing steps: Normalization: 1, intensity data were extracted from raw data (idat) files using an updated version of the pipeline [15]; 2, quantil normalization: intensity data were quantile normalized within subsets of probe types prior to converting to beta values. Samples were excluded in case of insufficient DNA quality, insufficient bisulfite conversion or failure in detection (detection P-value > 0.01 at more than 1% of positions), or sex-mismatch between phenotype and methylation data.

QC steps: 1, detection p-value threshold (positions/sites) > 0.01; 2, call rate < 95% samples; 3, exclusion of sex chromosomes; 4, accounting for batch effects: first 10 principle components of the control probes; 5, cell counts using Salas LA et al [4].

POSEIDON Cohort contains two datasets in this study, measured by 450K and EPIC arrays respectively.

ALSPAC cohort:

Cohort Description: The Avon Longitudinal Study of Parents and Children (ALSPAC) is a prospective pregnancy cohort study, which enrolled 14,541 pregnant women residing in Avon, United Kingdom who had expected delivery dates between April 1st, 1991 and December 31st, 1992. As described previously [16 17] detailed information has been collected on these participants and their offspring at regular intervals. Details of all data collected for ALSPAC are available through a fully searchable data dictionary, which is publicly available: http://www.bris.ac.uk/alspac/researchers/data-access/data-dictionary/. As part of the Accessible Resources for Integrated Epigenomic Studies (ARIES) project, DNA methylation was generated for 1018 mother-offspring pairs from the ALSPAC cohort, using the Infinium HumanMethylation450 BeadChip array (Illumina Inc., San Diego, United States). ARIES participants were selected based on availability of DNA samples at two time points for the mother (antenatal and at follow-up when the offspring were adolescents) and at three time points for the offspring (neonatal, childhood (age 7), and adolescence (age 15/17)).

Ethical approval: Ethical approval for the study was obtained from the ALSPAC Ethics and Law Committee and the Local Research Ethics Committees. Consent for biological samples

has been collected in accordance with the Human Tissue Act (2004). Informed consent for the use of data collected via questionnaires and clinics was obtained from the participants following the recommendations of the ALSPAC Ethics and Law Committee at the time.

Methods for methylation measurements in ALSPAC have been described previously [18]. See below for a brief description.

Blood collection and DNA extraction: Cord blood was collected according to standard procedures. DNA methylation assays and data pre-processing were performed at the University of Bristol as part of the ARIES project. DNA was extracted using standard protocol and was bisulfite-converted using the Zymo EZ DNA MethylationTM kit (Zymo, Irvine, CA). DNA methylation was then measured using the Infinium HM450 BeadChip assay (Illumina Inc, San Diego, CA), according to the standard protocol. Arrays were scanned using an Illumina iScan. An initial review of data quality was assessed using GenomeStudio (version 2011.1). A semi-random approach (sampling criteria were in place to ensure that all time points were represented on each array) was used to distribute ARIES samples across slides to minimize the possibility of potential confounding by batch.

Preprocessing steps: Data were normalised using the meffil R package [19] using the functional normalisation approach. Blood cell type proportions was estimated using the "Salas" reference set in the ''FlowSorted.CordBlood.Combined.450K'' Bioconductor package for cell type correction and normalised using meffil R package [19]. QC steps included removal of sex chromosomes, SNPs and control probes with a detection p-value over 0.05 for over 5% of samples.

MoBa 1 & 2 cohort

Mother-offspring pairs were selected from a sub-study within the Norwegian Mother, Father and Child Cohort Study (MoBa) [20 21]. The substudy was a cohort random sample and asthma cases at age three years [22]. Offspring in this study were born in 2002 to 2004 for children in MoBa1 and 2000-2005 for MoBa2.

MoBa 1&2 were approved by the Regional Committee for Ethics in Medical Research in Norway and the Institutional Review Board of the National Institute of Environmental Health Sciences in the USA. These analyses were analyzed using data release version 12.

Blood collection and DNA extraction: Methylation was measured in MoBa1 first (Joubert et al. 2012). The same laboratory measured DNA methylation in MoBa2 later (Joubert et al. 2016b). Details of how DNA methylation were measured and the quality control procedure for MoBa1 and MoBa2 have been previously described (Joubert et al. 2016b). The same procedures were applied to both studies. Briefly, samples of umbilical cord blood were collected at birth and stored at -80°C. DNA was bisulfite converted using the EZ-96 DNA Methylation kit (Zymo Research Corporation, Irvine, CA). DNA methylation was assessed at 485,577 CpGs using Illumina's Infinium HumanMethylation450 BeadChip (Bibikova et al. 2011). We used the minfi package in R to read the .idat files and calculated the beta methylation values at each CpG.

Preprocessing steps: Quality control procedures were applied on the beta methylation values. We excluded 65 control probes, probes on the X chromosome (# CpGs =11,230) and probes on the Y chromosome (# CpGs =416). CpGs missing >10% of methylation values were removed (20 CpGs in MoBa1, 0 CpGs in MoBa2). We excluded samples identified by Illumina to

have failed or those with an average detection p-value <0.05 across all probes (49 in MoBa1, 35 in MoBa2), as well as samples with sex mismatches (13 in MoBa1, 8 in MoBa2). We used the beta mixture quantile (BMIQ) to normalize the data [23] and used ComBat from the sva package in R for batch correction [24]. Beta methylation values were winsorized to the top and bottom 0.5% values.

Women were asked to report their earlier pregnancies, including pregnancies that ended in abortion, miscarriage, or stillbirth, the week of pregnancy that the abortion or stillbirth occurred, and ectopic pregnancies. For this study, we defined stillbirth based on >=20 weeks gestation.

Funding: The study was partly funded by the Research Council of Norway (project no. 320656, and through its Centres of Excellence funding scheme, project no 262700). The Norwegian Mother, Father and Child Cohort Study is supported by the Norwegian Ministry of Health and Care Services and the Ministry of Education and Research. We are grateful to all the participating families in Norway who take part in this on-going cohort study. Generation of the methylation data for MoBa1 and MoBa2 was supported by the Intramural Research Program of the NIH, National Institute of Environmental Health Sciences (project number ZO1 ES49019). The work was also partially supported by NIH/NIEHS contract no. N01-ES-75558.

MoBa 3 cohort

Mother-offspring pairs were selected from a sub-study within the MoBa Study[25]. Surrogate variable analysis (SVA) was performed on the methylome data to correct for potential batch effects, to adjust for differences in cell type composition as a reference-free method[26]. When considering specific selection factors, nested Case/Control status was utilized as the selection variable.

MoBa3 DNA methylation assays were funded by the grant from Institut National du Cancer (INCa) / INSERM-Plan Cancer (France, 2015) and the International Childhood Cancer Cohort Consortium (I4C). MoBa3 was approved by the Regional Committee for Ethics in Medical Research in Norway and the IARC Ethics Committee.

Preprocessing steps: Normalization using Funnorm. QC steps: 1. Boxplot colour: boxplot for both methylated and unmethylated data to identify outliers and assess sample qualityTwo samples failed this assessment. The distributions of raw data was already coherent across samples before normalization; 2. PlotQC to estimate sample-specific quality control (QC) for methylation data. One sample failed this QC check (one of the samples that also failed the boxplot colour analysis); 3. DensityPlot : The binomial distribution was evident in all samples and coherent across samples (except the two bad quality samples mentioned above); 4. MdsPlot : Sex was the major PC that segregated the data into two groups. Three samples were identified to have a sex mismatch using the getSex function and were hence excluded; 10 samples were excluded because they were not singletons, and 12 samples were excluded because they had NA values for many covariates. 5. Sex chromosomes, cross-reactive probes and SNPs were removed; 6. Probes with proportion of missing value higher than 10% were removed. The ones with missing value less than 10% were replaced by the mean.

Exclusions: Exclusions were done before running the SVA.

EXPOsOMICS

EXPOsOMICS is a population based European collaborative project which combined data from three birth cohorts: ENVIRONAGE (Belgium), Rhea (Greece) and Piccolipiu (Italy). Heterogeneity between the three cohorts was minimised by harmonising the phenotypic variables across the three cohorts and randomising the DNA samples on the DNA methylation arrays. Ethical approval for the Exposomics studies was obtained from IARC Ethics Committee as well as by the local ethics committees of each cohort as described below. We thank Dr. Paolo Vineis for coordinating the EXPOsOMICS project and Mr. Cyrille Cuenin and Mr. Vincent Cahais for their help in this project. The DNA methylation assays were funded by the European Community's Seventh Framework Programme FP7/2007–2013 project EXPOsOMICS (grant no. 308610).

ENVIR*ON*AGE cohort

Description of the ENVIR*ON*AGE cohort: ENVIR*ON*AGE (ENVIRonmental influence *ON* early AGEing) is an ongoing population-based prospective birth cohort study that aims at exploring human ageing and its interaction with the environment [27]. The cohort includes more than 2000 mother-infant pairs that have been recruited at the delivery at the East-Limburg Hospital in Genk (Belgium) from February 2010 onward. Inclusion criteria were delivery without planned caesarean section and ability to fill out a Dutch language questionnaire. Ethical approval was obtained by the Ethical Committee of Hasselt University and the East-Limburg Hospital and written informed consent was given by the parents. Data on lifestyle, diet, air pollution exposures, socio-economic status, phenotypic characteristics as well as clinical medical records and biological specimens have been collected at birth from mothers and children.

Preprocessing steps for 450K array: Funnorm. QC steps : 1. Boxplot colour : boxplot for meth and unmeth, to check for outliers and bad quality samples. The distributions of raw data was already coherent across samples before normalization; 2. PlotQC Estimate sample-specific quality control (QC) for methylation data; 3. DensityPlot : The binomial distribution was evident in all samples and coherent across samples; 4. MdsPlot : showed that the major PC that segregated the data into two groups was child sex. We identified two samples with sex mismatch using the getSex function and, hence, excluded those samples; 5. Sex chromosomes, cross-reactive probes and SNPs were removed; 6. We removed probes with a proportion of missing values being higher than 10%. The ones with missing values less than 10% were replaced by the mean. Exclusions were done before running the SVA. Two exclusions were due to potential sex mismatches, nine exclusions due to non-European ethnicity, and two exclusions due to missing values for Miscarriage/abortion. Exclusion of samples: Of the initially 372 samples with methylation data, 22 were excluded because they were also analyzed with the Illumina Infinium EPIC array, one sample had no information on maternal smoking status during pregnancy, three samples were removed because of siblings in the study population. Of the remaining samples, 20 originated from newborns of a non-European ancestry which brought the final sample size to 326.

Preprocessing steps for EPIC array: In a subset of 377 children from the ENVIR*ON*AGE cohort cord blood DNA was extracted from buffy coat according to standard protocol. DNA

concentration was measured using the Quant-IT assay from Thermo Fisher. Gel-electrophoresis was performed to assess the integrity of DNA samples. Methylation data was produced at GenomeScan in the Netherlands. Bisulphite conversion using 100-500 ng genomic DNA inputs was performed using the EZ DNA Methylation Gold kit (Zymo Research, Irvine, CA, USA). The converted samples (4µl) were amplified and hybridized on the Illumina HumanMethylation 850K BeadChip arrays and scanned using an Illumina iScan. Data quality was assessed using the R script MethylAid using analysis default settings and 1 sample was removed because of low quality (sample call rate <99%). DNA methylation data were preprocessed using the minfi package in R [28]. Briefly, 4 samples were removed because of inaccurately predicted sex using shinyMethyl [29], the data were normalized using functional normalization, measurements with detection p-values >10e-16 [15] were set to missing leading to removal of 7961 probes with a call rate <95%. No additional sample had a call rate <98%. For each CpG site methylation levels were expressed as beta values calculated as the ratios of intensities arising from methylated probes over those arising from the sum of methylated and unmethylated probes. Batch effect was corrected using the Combat algorithm [24]. After filtering the probes of the X and Y chromosomes and excluding probes overlapping with SNPs, 829,049 CpGs measured for 372 samples of the present analysis remained. Additionally, beta values were winsorized for the top and bottom 0.5% values.

Definition of exposures and covariates: At delivery the following information of parents and children were collected using questionnaires completed by the mothers: maternal smoking status during pregnancy (smoking during pregnancy/no smoking during pregnancy); ethnicity was considered to be European when two or more grandparents of the newborn were European and otherwise as non-European. Data derived from obstetric records were: parity (including live-births and still births), number of pregnancies (including additionally previous miscarriages and abortions), maternal age, pre-pregnancy weight, maternal weight gain during pregnancy, sex of the child, birthweight and gestational age (based on ultrasound). Cell type composition was estimated using filtered and combined reference datasets available via Bioconductor as "FlowSorted.CordBloodCombined.450 k" [4]. Each model was additionally adjusted for position on the array.

ENVIR*ON*AGE Cohort contains two datasets in this study, measured by 450K and EPIC arrays respectively.

Rhea cohort

The Mother-Child Cohort in Crete, Greece (Rhea Study) is a prospective birth cohort study of women (Greek and immigrants) who were pregnant between 2007 and 2008 and their children(29). The Rhea cohort was created in response to the rising concerns about the high rates of childhood obesity in Greece. Its primary objectives include: (1) to characterize nutritional, environmental and psychosocial determinants of children's growth and development; (2) to focus on four primary outcome areas of research:(i) offspring growth and obesity; (ii) neuropsychological and behavioral development; (iii) allergies and asthma in childhood; and (iv) genotoxicity; (3) to evaluate mother's health during and after pregnancy; (4) and to evaluate the interaction between environmental stressors and genetic variants in children's growth and health.

The majority of the cohort comprises individuals of European ethnicity, but this information was missing for some samples.
Surrogate variable analysis (SVA) was performed on the methylome data to correct for potential batch effects, to adjust for differences in cell type composition as a reference-free method [26].

Preprocessing steps: The data were normalized using the FunNorm function of the Bioconductor Minfi package [28]. QC steps: 1. Boxplot colour: boxplot for both methylated and unmethylated data to identify outliers and assess sample quality. The distributions of raw data was already coherent across samples before normalization; 2. PlotQC to estimate sample-specific quality control (QC) for methylation data; 3. DensityPlot : The binomial distribution was evident in all samples and coherent across samples; 4. MdsPlot : Sex was the major PC that segregated the data into two groups. We identified 6 samples with sex mismatch using the getSex function and hence excluded those samples; 5. Sex chromosomes, cross-reactive probes and SNPs were removed; 6.Probes with proportion of missing value higher than 10% were removed. The ones with missing value less than 10% were replaced by the mean.

Exclusions: Exclusions were done before running the SVA.

Piccolipiù Cohort

Piccolipiù is an Italian prospective birth cohort set up to investigate the effects of environmental exposures, parental conditions and social factors experienced during pre-natal and early post-natal life on infant and child health and development (28).

The majority of the cohort comprises individuals of European ethnicity, but this information was missing for some samples.

Surrogate variable analysis (SVA) was performed on the methylome data to correct for potential batch effects, to adjust for differences in cell type composition as a reference-free method[26].

Preprocessing steps: The data were normalized using the FunNorm function of the Bioconductor Minfi package [28]. QC steps: 1. Boxplot colour: boxplot for both methylated and unmethylated data to identify outliers and assess sample quality . The distributions of raw data was already coherent across samples before normalization; 2. PlotQC to estimate sample-specific quality control (QC) for methylation data; 3. DensityPlot: The binomial distribution was evident in all samples and coherent across samples;4. MdsPlot: Sex was the major PC that segregated the data into two groups. One sample was identified to have a sex mismatch using the getSex function and was hence excluded; 5. Sex chromosomes, cross-reactive probes and SNPs were removed; 6. Probes with proportion of missing value higher than 10% were removed. The ones with missing value less than 10% were replaced by the mean.

Exclusions: Exclusions were done before running the SVA.

PREDO cohort

The Prediction and Prevention of Preeclampsia and Intrauterine Growth Restriction (PREDO) [32] is a prospective birth cohort study of Finnish women who were pregnant between 2005 and 2010 and their children. The PREDO study cohort was set up to identify novel risk factors and biomarkers in pregnant women associated with the development of preeclampsia and intrauterine growth restriction (IUGR), to (a) identify effective methods for prediction and

prevention of preeclampsia in at-risk women, and (b) determine the association between exposure to preeclampsia, IUGR, or their risk factors and child developmental/health outcomes. Women with a singleton, intrauterine pregnancy who visited antenatal clinics at ten study hospitals in Finland for their first ultrasound screening at 12+0-13+6 weeks+days of gestation were recruited in the PREDO study. Two groups of pregnant women were enrolled: first, pregnant women with a known clinical risk factor status for preeclampsia and IUGR, and second, pregnant women who volunteered to participate regardless of their risk factor status for preeclampsia and IUGR. The sample with a known risk factor status comprises 1,079 pregnant women who gave live birth (969 of these women had at least one and 110 had none of the known risk factors for preeclampsia and IUGR). The community-based sample comprises 3,698 pregnant women who gave live birth. The sample with a known risk factor status visited antenatal clinics up to four times during pregnancy and both samples filled in bi-weekly self-reports. The post-delivery follow-up has taken place at approximately 2 weeks, 6 months, and 3.5 years after the delivery. The most recent follow-up started in 2016 and is ongoing. The study protocol was approved by the Ethics Committee of Obstetrics and Gynaecology, and Women, Children and Psychiatry of the Helsinki and Uusimaa Hospital District and by the participating hospitals (Dnro 5/E8/05, Dnro 3/E8/05, 216/13/03/03/2012). All participants provided written informed consent. Consent of participating children were provided by parent(s)/guardian(s). Details of the study design, inclusion criteria, enrollment and data collection are described elsewhere (1). The study has been registered as ClinicalTrials.gov identifier ISRCTN14030412.

The PREDO Study has been funded by the Academy of Finland (JL: 311617 and 269925, KR: 1312670 ja 128789 1287891), EraNet Neuron, EVO (a special state subsidy for health science research), University of Helsinki Research Funds, the Signe and Ane Gyllenberg foundation, the Emil Aaltonen Foundation, the Finnish Medical Foundation, the Jane and Aatos Erkko Foundation, the Novo Nordisk Foundation, the Päivikki and Sakari Sohlberg Foundation, Juho Vainio foundation, Yrjö Jahnsson foundation, The Finnish Society of Sciences and Letters, Jalmari and Rauha Ahokas foundation, Sigrid Juselius Foundation granted to members of the Predo study board. Methylation assays were funded by the Academy of Finland (269925).

The PREDO study would not have been possible without the dedicated contribution of the PREDO study group members: E Hamäläinen, E Kajantie, H Laivuori, PM Villa, A-K Pesonen, A Aitokallio-Tallberg, A-M Henry, VK Hiilesmaa, T Karipohja, R Meri, S Sainio, T Saisto, S Suomalainen-Konig, V-M Ulander, T Vaitilo (Department of Obstetrics and Gynaecology, University of Helsinki and Helsinki University Central Hospital, Helsinki, Finland), L Keski-Nisula, Maija-Riitta Orden (Kuopio University Hospital, Kuopio Finland), E Koistinen, T Walle, R Solja (Northern Karelia Central Hospital, Joensuu, Finland), M Kurkinen (Päijät-Häme Central Hospital, Lahti, Finland), P.Taipale. P Staven (Iisalmi Hospital, Iisalmi, Finland), J Uotila (Tampere University Hospital, Tampere, Finland). We thank all the PREDO children and their parents for their enthusiastic participation. We also thank all the research nurses, research assistants, and laboratory personnel involved in the Predo study.

Blood collection and DNA extraction: In the high-risk sample, cord blood samples were collected according to standard procedures. DNA was extracted at the National Institute for Health and Welfare, Helsinki, Finland and and the Finnish Institute of Molecular Medicine, University of Helsinki, Finland and methylation analyses were performed at the Max Planck

Institute in Munich, Germany. Methylation analyses were performed at the Max Planck Institute of Psychiatry in Munich, Germany. DNA was bisulphite-converted using the EZ-96 DNA Methylation kit (Zymo Research, Irvine, CA). Samples were ran on Illumina 450K Methylation arrays and the arrays were scanned using the iScan System (Illumina Inc., San Diego, CA).

Preprocessing steps: The quality control pipeline pipeline was set up using the R-package minfi. 3 IDs were excluded as they were outliers in the median intensities. Furthermore, 20 IDs showed disconcordance between phenotypic sex and estimated sex and were excluded. Methylation beta-values were normalized using the funnorm function. We excluded any probes on chromosome X or Y, probes containing SNPs and cross-hybridizing probes. Furthermore, any Cgs with a detection p-value > 0.01 in at least 50% of the samples were excluded. The final dataset contains 428,619 CpGs and 834 IDs. We used ComBat to check and adjust for the batch effects (slide and well). Cord blood cell counts were estimated for seven cell types (nucleated red blood cells, granulocytes, monocytes, natural killer cells, B cells, CD4(+)T cells, and CD8(+)T cells) using the method of Bakulski et al [4], which is incorporated in the R-package *minfi.*

PREDO Cohort contains two datasets in this study, measured by 450K and EPIC arrays respectively.

HERO-G cohort

The Gambian HERO-G (Hormonal and Epigenetic Regulators of Growth) study is a prospective cohort study from rural Gambia, West Africa that followed mothers and children longitudinally from pre-conception, through pregnancy, delivery, and to two years of child age [33]. The primary focus of the study is to explore mechanistic pathways influencing patterns of growth in early infancy.

Blood collection and DNA extraction: 136 samples cord blood samples were processed on the EPIC array.

Preprocessing steps: Methylation calls were generated from EPIC IDATs using the *meffil* pipeline (v1.1.1) with default parameters for quality control (QC) and normalisation [19]. Briefly, 18 samples were removed following QC, 16 samples with >20% failed probes and 2 sex mismatches; and 927 CoGs were removed that failed in >20% samples and/or had <3 beads. Following QC, data for 864,932 CpGs measured in 118 samples were available for normalisation. The m*effil* normalisation pipeline performed dye bias and background correction of probe intensities using the 'noob' method, followed by quantile normalisation based on the top 10 principal components from a PCA of control probe intensities [19].

Three further samples with missing data on birthweight and gestational age were removed leaving a total of n=115 samples with 864,932 CpGs available for analysis. Stillbirth for previous pregnancies was defined as pregnancy loss after 28 weeks. Gestational age at birth was determined from ultrasound scans carried out on women at the time pregnancy was confirmed. Note that female smoking is very rare in this population, and this is therefore not included as a covariate in this analysis.

Finally, adjustment for technical / batch effects was carried out by including the top 5 principal components from a PCA of the normalised methylation beta values in all regression models. These were significantly associated with sample plate, slide and sentrix row. No other known technical variables were associated with any of the top 10 PCs.

INMA Cohort

The INMA—INfancia y Medio Ambiente— (Environment and Childhood) Project is a network of birth cohorts in Spain that aim to study the role of environmental pollutants in air, water and diet during pregnancy and early childhood in relation to child growth and development. In this study data on the cohort set up in Sabadell (Catalonia, Spain) between 2004 and 2008 was evaluated, further details explained elsewhere [34]. INMA Sabadell study has 622 children at birth, however cord blood DNA methylation was only available for 391 children, and only 383 individuals had birth order measurements. In those models excluding miscarriage/abortion a total of 290 individuals were assessed. For those models including miscarriage/abortion, a total of 383 inidividuals were assessed. Considering just complete cases (no missing in any of the covariates used) a final sample of 285 individuals was evaluated excluding miscarriage/abortion and 377 individuals was evaluated including miscarriage/abortion. For the sensitivity analyses a total of 285 individuals were assessed including pre-pregnancy weight of mother as an additional covariate and 281 individuals were assessed including pregnancy weight gain of mother as an additional covariate.

Blood collection and DNA extraction**:** Cord blood was extracted using the Chemagen kit (Perkin Elmer). DNA concentration was determined by NanoDrop spectrophotometer (Thermo Scientific) and with the Quant-iT PicoGreen dsDNA Assay Kit (Life Technologies). Methylation data was produced in two different laboratories as part of two different projects: in the Genome Analysis Facility of the University Medical Center Groningen (UMCG) in Holland, and in the Bellvitge Biomedical Research Institute (IDIBELL, Barcelona). Both laboratories used the recommended Illumina protocol for the Infinium HumanMethylation450 beadchip. Briefly, 500 ng of DNA was bisulfite-converted using the EZ 96-DNA methylation kit following the manufacturer’s standard protocol, and DNA methylation measured using the Illumina Infinium HumanMethylation450 beadchip. DNA methylation data were preprocessed using the minfi package [28].

Preprocessing steps: A series of steps were completed for quality control and data analysis. The first step was low quality sample removal. First, 2 samples with bad overall quality or with low detection p-value according to the output of the MethylAid package [35] were removed. Then, we removed 3 samples whose sex was wrongly predicted using shinyMethyl [36]. Following guidelines of Lehne [15] work, we increased the stringency of the detection p-value threshold to 10e-16 and we filtered 18 samples with a call rate lower than 98%. The second step was normalizing data with functional normalization. Correlation between SNP in replicates samples was checked and probes not measuring SNPs were discarded. 7,136 probes with a call rate lower than 95% were also removed. Probes in sexual chromosomes, crosshibridizing or containing SNPs were flagged but not removed at this point. ComBat was applied to remove batch effect [24]. Finally, duplicated samples were removed, prioritizing MeDALL samples over BREATHE samples. The final dataset consisted of 391 at age 0y and 476,946 probes. For the current study we used European ancestry children.

Stillbirth in INMA refers to fetal death 22 weeks of pregnancy and later.

Generation R Study

Description of cohort: Generation R Study is a population-based prospective cohort study from fetal life onwards established in Rotterdam, the Netherlands [37]. Pregnant women with an expected delivery date between April 2002 and January 2006 living in Rotterdam were eligible to participate, and written informed consent was obtained from all participants. In 1396 of the 9901 live-born newborns participating in the Generation R Study, we measured genome-wide DNA methylation in cord blood. This subgroup was selected from the total study population as a relatively homogeneous, European-ancestry subgroup. A total of 11 mothers had two (non-twin) children. Per mother, we included only one child, and the selection was random. In our cohort there was no information on older siblings being twins or singletons. As the vast majority would be singletons, we did not apply any selection on this. In the current analysis, we included mother–newborn pairs who had complete information on the exposure and covariate variables, a total of 1249 participants.

Birth order: ordinal variable created based on maternal parity, which was collected from pregnancy questionnaires. The 5th and 4th born children were merged into the same category, due to small number of 5th born children (N=1).

Blood collection and DNA extraction: We used the salting-out method to extract DNA from cord blood samples. Five-hundred nanograms of DNA were bisulfite converted using the EZ-96 DNA Methylation kit (Shallow) (Zymo Research Corporation, Irvine, USA). Samples were processed with the Illumina Infinium HumanMethylation450 BeadChip (Illumina Inc., San Diego, USA).

Preprocessing steps: Quality control and normalization were performed using the CPACOR workflow [15]. Probes with a detection $p \geq 1E-16$ were set to missing. Intensity values were quantile normalized. We removed arrays with technical problems, a call rate ≤ 95%, or a mismatch between the expected sex of participant and sex determined by chromosome X and Y probe intensities. Probes on the sex chromosomes were removed before the analyses. We used untransformed beta-values as measures of DNA methylation. The final DNA methylation dataset contained information on 458,563 CpGs.

Covariates: Child sex (male=1, female =0) and birth weight were obtained from midwife and hospital registries. Participating pregnant mothers were seen in the first trimester of pregnancy for fetal ultrasound at our research center and gestational age was determined during this visit [38]. If mothers had a known and reliable first day of the last menstrual period, and a regular menstrual cycle of 28 ± 4 days, the clinical estimate of gestational age was based on that. If mothers did not know the exact date of their last menstrual period, or had an irregular menstrual cycle, we established gestational age by ultrasound examination.

Maternal age was reported by the mother at intake. Maternal smoking during pregnancy was self-reported as “no smoking during pregnancy”, “smoked but quit before second trimester”, or “smoked throughout pregnancy”. We recoded this variable into two dummy variables: (1) no smoking versus any smoking, and (2) no smoking or sustained smoking versus quit smoking in early pregnancy. Maternal early-pregnancy was measured at the research center at intake. This variable was used instead of pre-pregnancy self-reported weight, due to a high correlation between the two, and less missing information in the variables measured at the center. Maternal weight was measured again in late pregnancy, 3rd trimester visit, around 30 weeks of gestation. Pregnancy weight gain was calculated subtracting early-pregnancy weight to 3rd trimester weight.

Plate number was included as to account for batch effects. Cell type proportions were estimated based on the Salas et al cord blood panel. This method estimates the proportions of white blood cell subtypes CD8+ T cells, CD4+ T cells, natural killer cells, B cells, monocytes, granulocytes, and nucleated red blood cells in cord blood (Salas et al 2019).

Acknowledgements: The Generation R Study is conducted by Erasmus MC, University Medical Center Rotterdam in close collaboration with the School of Law and Faculty of Social Sciences of the Erasmus University Rotterdam, the Municipal Health Service Rotterdam area, Rotterdam, the Rotterdam Homecare Foundation, Rotterdam and the Stichting Trombosedienst & Artsenlaboratorium Rijnmond (STAR-MDC), Rotterdam. We gratefully acknowledge the contribution of children and parents, general practitioners, hospitals, midwives and pharmacies in Rotterdam. The study protocol was approved by the Medical Ethical Committee of the Erasmus Medical Centre, Rotterdam. Written informed consent was obtained for all participants. The generation and management of the Illumina 450K methylation array data (EWAS data) for the Generation R Study was executed by the Human Genotyping Facility of the Genetic Laboratory of the Department of Internal Medicine, Erasmus MC, the Netherlands. We thank Mr. Michael Verbiest, Ms. Mila Jhamai, Ms. Sarah Higgins, Mr. Marijn Verkerk and Dr. Lisette Stolk for their help in creating the EWAS database. We thank Dr. A.Teumer for his work on the quality control and normalization scripts.

Funding: The general design of the Generation R Study is made possible by financial support from the Erasmus MC, Erasmus University Rotterdam, the Netherlands Organization for Health Research and Development and the Ministry of Health, Welfare and Sport. The EWAS data were funded by a grant to VWJ from the Netherlands Genomics Initiative (NGI)/Netherlands Organisation for Scientific Research (NWO) Netherlands Consortium for Healthy Aging (NCHA; project nr. 050-060-810), by funds from the Genetic Laboratory of the Department of Internal Medicine, Erasmus MC, and by a grant from the National Institute of Child and Human Development (R01HD068437). This project received funding from the European Union's Horizon 2020 research and innovation programme (733206, LifeCycle; 848158, EarlyCause; 874739, LongITools; 874583, ATHLETE; 824989, EUCAN-Connect) and from the European Joint Programming Initiative "A Healthy Diet for a Healthy Life" (JPI HDHL, NutriPROGRAM project, ZonMw the Netherlands no.529051022).

Finngedi cohort

Cohort description: This subcohort is a part of the Finnish Gestational Diabetes (FinnGeDi) Study including 299 women with gestational diabetes mellitus (GDM) and 238 controls aged 19–45 years [39]. Women with GDM were recruited as they came to give birth, and the next consenting woman without GDM was recruited as a control from seven delivery hospitals in Finland (Oulu, Helsinki, Jyväskylä, Pori, Kajaani, Seinäjoki and Lappeenranta) between 2/2009 and 12/2012. Women with prepregnancy diabetes and multiple pregnancy were excluded. GDM was diagnosed by 2-hour 75 g oral glucose tolerance test (OGTT) (at 5-37 weeks of gestation) where the cut-off concentrations for venous plasma glucose were ≥ 5.3 mmol/l at baseline (fasting glucose), ≥ 10.0 mmol/l at 1 h or ≥ 8.6 mmol/l at 2 h after glucose intake. GDM diagnosis was set if one or more glucose concentrations exceeded the cut-off levels. Controls were confirmed by OGTT after 24 weeks of gestation. Data include cord blood DNA sample, clinical data from hospital and maternal welfare records, register data from

national registers, and self-reported lifestyle, medical and family history data from questionnaires. Cohort is located at the National Institute for Health and Welfare (Oulu and Helsinki, Finland).

Preprocessing steps: IDATS were imported using functions in the *ChAMP* package [40]. Samples with call rate below 95 % and probes with call rate below 100 % were excluded. Call rates were computed using a 1 % threshold on the detection p-values. Probes which align to multiple locations, are non-CpG, with bead counts less than 3 were excluded. Type I and type II probes were normalised using the Regression on Correlated Probes (RCP) method [41] from the *ENmix* package [42]. Data were normalised for possible batch effect bias using the Combat function from the *sva* package, with batch/slide as covariate [24].

Specific variables: Covariates used in the analyses included: sex (binary, 0=female, 1=male), maternal age (continuous in years), gestational age at birth (continuous in weeks), stillbirth cut off 22+weeks, miscarriage and abortion cut off 22 weeks (21+6 weeks, 22+0 and after=stillbirth or delivery), selection factor (gestational diabetes status, binary, 0=control, 1=GDM). Cell proportions were estimated using estimateCellCounts() in the *minfi* package [28] and the reference panel *FlowSorted.Blood.EPIC* [43]. To improve the performance of this estimate, we used the IDentifying Optimal DNA methylation Libraries (IDOL) algorithm [8]. Cell estimates included in the models were nRBC, CD8T, CD4T, NK, Bcell, Mono and Gran. All mothers were non-smokers and of Finnish ancestry.

Isle of Wight birth cohort– F1 generation (IOWBC-F1)

The F1 generation of the IOW cohort is a Caucasian whole population birth cohort was established on the Isle of Wight, UK, in 1989 to prospectively study the natural history of allergies [38]. The local Research Ethics Committee approved the study. Informed consent was obtained from parents of 1456 newborns to enroll in the study who were born between 1 January 1989, and 28 February 1990. Children were followed up at the ages of 1 (n = 1167), 2 (n = 1174), 4 (n = 1218), and 10 (n = 1373) years. At each follow up questionnaires were completed for each child. If a visit was not possible, a telephone questionnaire was completed or a short postal questionnaire sent for completion and return.

Blood collection and DNA extraction: In the F1 generation, DNA was isolated from dried blood spots on Guthrie cards of 796 neonates using a method based on the procedure described by Beyan et al [44]. DNA concentration was determined by Qubit quantitation. One microgram of DNA was bisulfite-treated for cytosine to thymine conversion using the EZ 96-DNA methylation kit (Zymo Research, CA, USA), following the manufacturer's standard protocol. Epigenome-scale DNA methylation (DNAm) was assessed using the Illumina Infinium MethylationEPIC BeadChip (Illumina, Inc., San Diego, CA, USA), which interrogates > 850,000 CpGs associated with over 24,000 genes. The estimateCellCounts() function from Minfi package[8], with the adult reference panel was used for cell type estimation4 from heel prick DNAm.

Preprocessing steps: The CPACOR pipeline was used for quality control (QC) and pre-processing the quantile normalized beta values from the samples. ComBat[27] was applied to remove batch effects. CpG sites with probe-SNPs within ten base pairs and with minor allele frequency (MAF)

greater than 0.007 (which represented about 10 subjects in expectation in the complete study cohort) were excluded. This resulted finally in 551,710 CpGs from 796 participants.

Isle of Wight Birth Cohort – F2 Generation (IOWBC-F2)

Design and study population: The recruitment of new-borns started from April 2010. Data used in the analyses were from infants born between April 2010 to May 2014 [45]. In total, 200 new-borns were recruited such that at least one of their parents is in the IOW birth cohort (IOW F1) and recruitment is ongoing. For each infant, along with other phenotypic information such as gender and birthweight, status of wheezing and eczema was recorded, measures of wheal size from skin prick test as well as IgE were recorded.

Birth order: The birth order variables (gravida and para (Parity)) are taken from the maternity records, including all births (live or stillborn) and all pregnancies whether or not they resulted in a birth.

Blood collection and DNA extraction: For 130 subjects, we measured DNA methylation from whole blood processed with the Illumina Infinium HumanMethylation450 Beadchip (Illumina Inc., San Diego, USA).

Preprocessing steps: CPACOR [15] pipeline was used for QC and normalisation of the data. Methylation markers on 65 single nucleotide polymorphism (SNP) and sex chromosomes were removed. We applied Illumina background correction to all intensity values. Any intensity values having detection p-values >= 10^{-16} were set as missing data. Samples with call rate < 98% were excluded. A quantile normalisation was applied using limma on intensity values separately based on six different probe-type categories (Type-I M red, Type-I U red, Type-I M green, Type-I U green, Type-II red, and Type-II green). Beta values were then calculated from these normalised intensity values. After choosing only singletons, non-missing variable and the QC, 456,026 sites remain for the 104 samples for final analysis.

Covariates: Covariates were collected via questionnaires collected at recruitment, before and during pregnancy. Maternal age was derived from mothers' date of birth. Child's gender and gestational age at birth were collected from stored clinical information. Maternal smoking status was collected from the responses from the questionnaires. Maternal smoking status in pregnancy (Yes/No) was defined as any smoking in pregnancy or no smoking in pregnancy. Birth weight was collected from stored clinical records. The outliers for observe birth weight values +/- 5 SD from the mean was checked. Cell type correction: estimates based on the Salas et al. cord blood reference panel [43], nRBC, CD8T, CD4T, NK, Bcell, Mono, Gran. Batch correction: Indicator of different batches that DNA methylation data was collected and ComBat [24] was used to remove any batch effect.

Funding of the DNA methylation: The IoW 1989 (IOWBC) cohorts were supported by the National Institute of Allergy and Infectious Diseases and the National Heart, Lung, and Blood Institute under the award number R01 AI091905and R01 HL132321, respectively (PI: Wilfried Karmaus).

Acknowledgements: We would like to thank all the participants of the Isle of Wight birth cohorts, the research team at David Hide Asthma & Allergy Research Centre (Isle of Wight) for collecting the data. In particular, the nurses for their help in recruiting them, Stephen Porter, Sharon Matthews, Frances Mitchell, Nikki Graham for technical support and other members of

the IoW research group for valuable discussion. DNA methylation data was generated by the Oxford Genomics Centre at the Wellcome Trust Centre for Human Genetics.

IOW F2 Cohort contains two datasets in this study, measured by 450K and EPIC arrays respectively.

CCLS cohort

The California Childhood Leukemia Study (CCLS) is a population-based case-control study, designed to understand genetic and environmental risk factors for childhood leukemia. Cohort description see [46]. CCLS involved recruitment at multiple facilities. Cases are subjects diagnosed with ALL in 16 participating hospitals in California. Controls were from the California birth registry. The CCLS is funded in part by the National Institute of Environmental Health Sciences (NIEHS), the National Cancer Institute (NCI), and the US Environmental Protection Agency.

Blood collection and DNA extraction: Blood samples were extracted from leftover neonatal dried blood spots (DBS) from the California Newborn Screening program. DNA from approximately one fourth of a spot was then extracted from DBS using the Qiagen QIAamp DNA Micro Kit protocol.

Preprocessing steps: Initial QC was performed with Illumina GenomeStudio, data was then preprocessed using “minfi” package [28], using “preprocessFunnorm” function, with built-in noob normalization step. Probes and subjects with more than 5% missing values were excluded and the remaining missing values were imputed using “impute.knn” function from impute [47] R package. To estimate proportions of nucleated blood cells, Identifying Optimal Libraries (IDOL) algorithm [8] was used as covariates in the regression analysis.

CCLS Cohort analyses contains four datasets in this study, 2 phases of CCLS samples were collected at different times, and White and Latino subjects from each phase were run separately (after QC, Phase 1, 483,287 probes, 199 Whites, 170 Latinos; Phase 2, 484,552 probes, 148 Whites, 219 Latinos) to avoid heterogeneity caused by mixed population.

Supplementary Reference

1. Herberth, G. *et al.* Maternal immune status in pregnancy is related to offspring's immune responses and atopy risk: Immune status in pregnancy and offspring's immune responses. *Allergy* **66**, 1065–1074 (2011).
2. Hinz, D. *et al.* Cord blood Tregs with stable FOXP3 expression are influenced by prenatal environment and associated with atopic dermatitis at the age of one year. *Allergy* **67**, 380–389 (2012).
3. Bauer, T. *et al.* Environment-induced epigenetic reprogramming in genomic regulatory elements in smoking mothers and their children. *Mol. Syst. Biol.* **12**, 861 (2016).
4. Gervin, K. *et al.* Systematic evaluation and validation of reference and library selection methods for deconvolution of cord blood DNA methylation data. *Clin. Epigenetics* **11**, 125 (2019).
5. Stein, D. J. *et al.* Investigating the psychosocial determinants of child health in Africa: The Drakenstein Child Health Study. *J. Neurosci. Methods* **252**, 27–35 (2015).
6. Fortin, J.-P. *et al.* Functional normalization of 450k methylation array data improves replication in large cancer studies. *Genome Biol.* **15**, 503 (2014).
7. Leek, J. T., Johnson, W. E., Parker, H. S., Jaffe, A. E. & Storey, J. D. The sva package for removing batch effects and other unwanted variation in high-throughput experiments. *Bioinformatics* **28**, 882–883 (2012).
8. Koestler, D. C. *et al.* Improving cell mixture deconvolution by identifying optimal DNA methylation libraries (IDOL). *BMC Bioinformatics* **17**, 120 (2016).

9. Du, P., Kibbe, W. A. & Lin, S. M. lumi: a pipeline for processing Illumina microarray. *Bioinforma. Oxf. Engl.* **24**, 1547–1548 (2008).
10. Hannum, G. *et al.* Genome-wide methylation profiles reveal quantitative views of human aging rates. *Mol. Cell* **49**, 359–367 (2013).
11. Efron, B., Turnbull, B., Narasimhan, B. & Strimmer, K. locfdr: Computes Local False Discovery Rates. (2015).
12. Schalkwyk, L. C. *et al.* wateRmelon: Illumina 450 and EPIC methylation array normalization and metrics. (2022) doi:10.18129/B9.bioc.wateRmelon.
13. Pidsley, R. *et al.* Critical evaluation of the Illumina MethylationEPIC BeadChip microarray for whole-genome DNA methylation profiling. *Genome Biol.* **17**, 208 (2016).
14. Price, M. E. *et al.* Additional annotation enhances potential for biologically-relevant analysis of the Illumina Infinium HumanMethylation450 BeadChip array. *Epigenetics Chromatin* **6**, 4 (2013).
15. Lehne, B. *et al.* A coherent approach for analysis of the Illumina HumanMethylation450 BeadChip improves data quality and performance in epigenome-wide association studies. *Genome Biol.* **16**, 37 (2015).
16. Boyd, A. *et al.* Cohort Profile: the ’children of the 90s’--the index offspring of the Avon Longitudinal Study of Parents and Children. *Int. J. Epidemiol.* **42**, 111–127 (2013).
17. Fraser, A. *et al.* Cohort Profile: the Avon Longitudinal Study of Parents and Children: ALSPAC mothers cohort. *Int. J. Epidemiol.* **42**, 97–110 (2013).
18. Relton, C. L. *et al.* Data Resource Profile: Accessible Resource for Integrated Epigenomic Studies (ARIES). *Int. J. Epidemiol.* **44**, 1181–1190 (2015).

19. Min, J. L., Hemani, G., Davey Smith, G., Relton, C. & Suderman, M. Meffil: efficient normalization and analysis of very large DNA methylation datasets. *Bioinforma. Oxf. Engl.* **34**, 3983–3989 (2018).
20. Magnus, P. *et al.* Cohort profile: The Norwegian Mother and Child Cohort Study (MoBa). *Int. J. Epidemiol.* **35**, 1146–1150 (2006).
21. Rønningen, K. S. *et al.* The biobank of the Norwegian Mother and Child Cohort Study: a resource for the next 100 years. *Eur. J. Epidemiol.* **21**, 619–625 (2006).
22. Håberg, S. E. *et al.* Maternal folate levels in pregnancy and asthma in children at age 3 years. *J. Allergy Clin. Immunol.* **127**, 262–264, 264.e1 (2011).
23. Teschendorff, A. E. *et al.* A beta-mixture quantile normalization method for correcting probe design bias in Illumina Infinium 450 k DNA methylation data. *Bioinformatics* **29**, 189–196 (2013).
24. Johnson, W. E., Li, C. & Rabinovic, A. Adjusting batch effects in microarray expression data using empirical Bayes methods. *Biostat. Oxf. Engl.* **8**, 118–127 (2007).
25. Joubert, B. R. *et al.* DNA Methylation in Newborns and Maternal Smoking in Pregnancy: Genome-wide Consortium Meta-analysis. *Am. J. Hum. Genet.* **98**, 680–696 (2016).
26. Kaushal, A. *et al.* Comparison of different cell type correction methods for genome-scale epigenetics studies. *BMC Bioinformatics* **18**, 216 (2017).
27. Janssen, B. G. *et al.* Cohort Profile: The ENVIRonmental influence ON early AGEing (ENVIRONAGE): a birth cohort study. *Int. J. Epidemiol.* **46**, 1386–1387m (2017).
28. Aryee, M. J. *et al.* Minfi: a flexible and comprehensive Bioconductor package for the analysis of Infinium DNA methylation microarrays. *Bioinformatics* **30**, 1363–1369 (2014).

29. Fortin, J.-P., Fertig, E. & Hansen, K. shinyMethyl: interactive quality control of Illumina 450k DNA methylation arrays in R. *F1000Research* **3**, 175 (2014).

30. Chatzi, L. *et al.* Cohort Profile: The Mother-Child Cohort in Crete, Greece (Rhea Study). *Int. J. Epidemiol.* **46**, 1392–1393k (2017).

31. Farchi, S. *et al.* Piccolipiù, a multicenter birth cohort in Italy: protocol of the study. *BMC Pediatr.* **14**, 36 (2014).

32. Girchenko, P. *et al.* Cohort Profile: Prediction and prevention of preeclampsia and intrauterine growth restriction (PREDO) study. *Int. J. Epidemiol.* **46**, 1380–1381g (2017).

33. Moore, S. E. *et al.* Identification of nutritionally modifiable hormonal and epigenetic drivers of positive and negative growth deviance in rural African fetuses and infants: Project protocol and cohort description. *Gates Open Res.* **4**, 25 (2020).

34. Guxens, M. *et al.* Cohort Profile: The INMA—INfancia y Medio Ambiente—(Environment and Childhood) Project. *Int. J. Epidemiol.* **41**, 930–940 (2012).

35. van Iterson, M. *et al.* MethylAid: Visual and interactive quality control of large Illumina 450k data sets. *Bioinforma. Oxf. Engl.* **30**, 3435–3437 (2014).

36. Fortin, J. P., Fertig, E. & Hansen, K. shinyMethyl: Interactive quality control of Illumina 450k DNA methylation arrays in R. *F1000Research* **3**, (2014).

37. Kooijman, M. N. *et al.* The Generation R Study: design and cohort update 2017. *Eur. J. Epidemiol.* **31**, 1243–1264 (2016).

38. Gaillard, R., Steegers, E. A., de Jongste, J. C., Hofman, A. & Jaddoe, V. W. Tracking of fetal growth characteristics during different trimesters and the risks of adverse birth outcomes. *Int. J. Epidemiol.* **43**, 1140–1153 (2014).

39. Keikkala, E. *et al.* Cohort Profile: The Finnish Gestational Diabetes (FinnGeDi) Study. *Int. J. Epidemiol.* **49**, 762–763g (2020).

40. Tian, Y. *et al.* ChAMP: Chip Analysis Methylation Pipeline for Illumina HumanMethylation450 and EPIC. (2022) doi:10.18129/B9.bioc.ChAMP.

41. Niu, L., Xu, Z. & Taylor, J. A. RCP: a novel probe design bias correction method for Illumina Methylation BeadChip. *Bioinformatics* **32**, 2659–2663 (2016).

42. Xu, Z., Niu, L. & Taylor, J. ENmix: Quality control and analysis tools for Illumina DNA methylation BeadChip. (2022) doi:10.18129/B9.bioc.ENmix.

43. Salas, L. A. *et al.* An optimized library for reference-based deconvolution of whole-blood biospecimens assayed using the Illumina HumanMethylationEPIC BeadArray. *Genome Biol.* **19**, 64 (2018).

44. Beyan, H. *et al.* Guthrie card methylomics identifies temporally stable epialleles that are present at birth in humans. *Genome Res.* **22**, 2138–2145 (2012).

45. Arshad, S. H. *et al.* Cohort Profile Update: The Isle of Wight Whole Population Birth Cohort (IOWBC). *Int. J. Epidemiol.* **49**, 1083–1084 (2020).

46. Whitehead, T. P. *et al.* Concentrations of persistent organic pollutants in California women's serum and residential dust. *Environ. Res.* **136**, 57–66 (2015).

47. Hastie, T., Tibshirani, R., Narasimhan, B. & Chu, G. impute: impute: Imputation for microarray data. (2022) doi:10.18129/B9.bioc.impute.

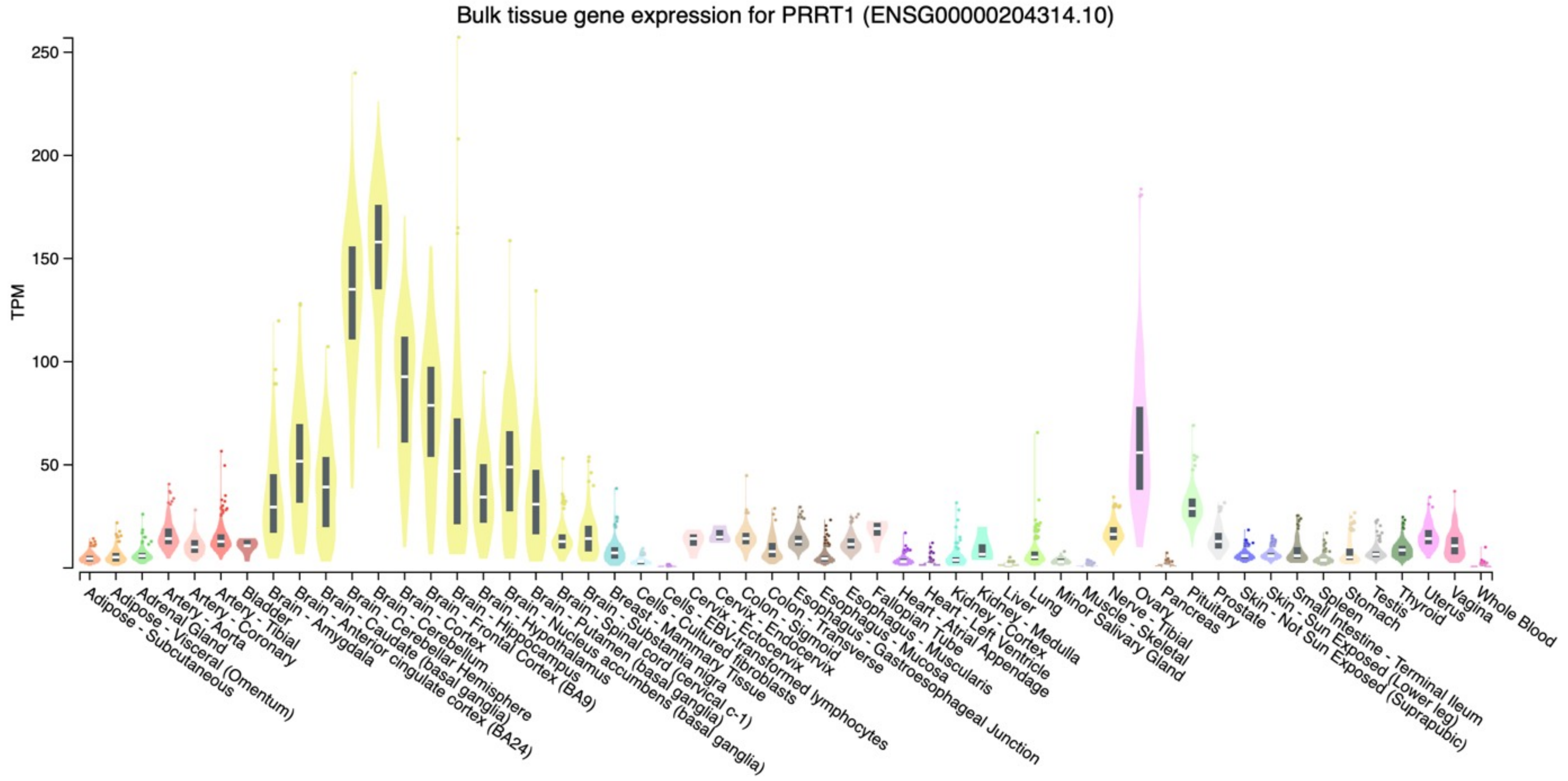

Supplementary Figure 1: PRRT1 expression profiles across tissues. TPM: transcript per million. Violin plots show full range, and box plots nested inside show 25% percentile (lower limit), median (middle line) and 75% percentile (higher limit).

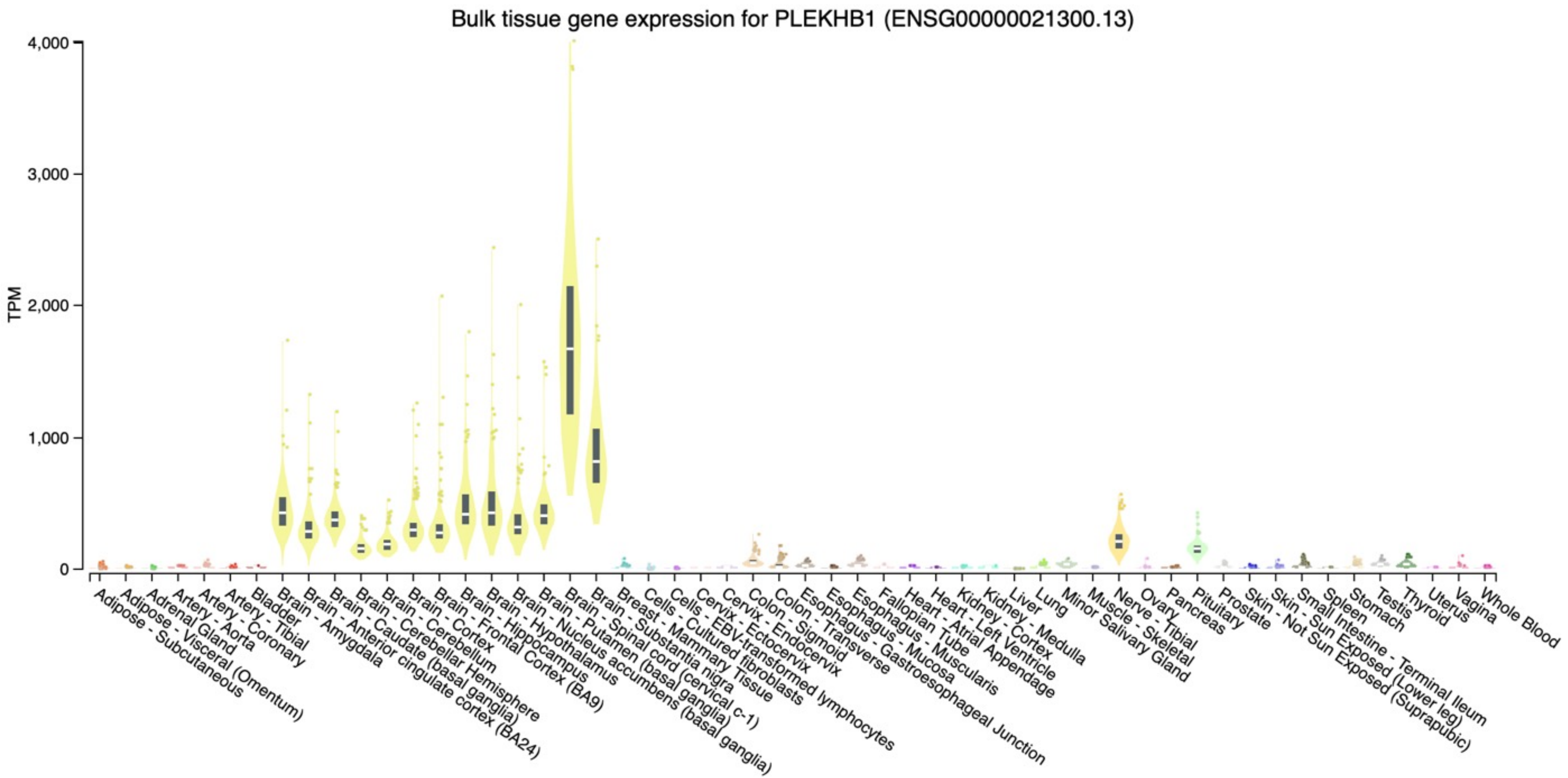

Supplementary Figure 2: PLEKHB1 expression profiles across tissues. Violin plots show full range, and box plots nested inside show 25% percentile (lower limit), median (middle line) and 75% percentile (higher limit).

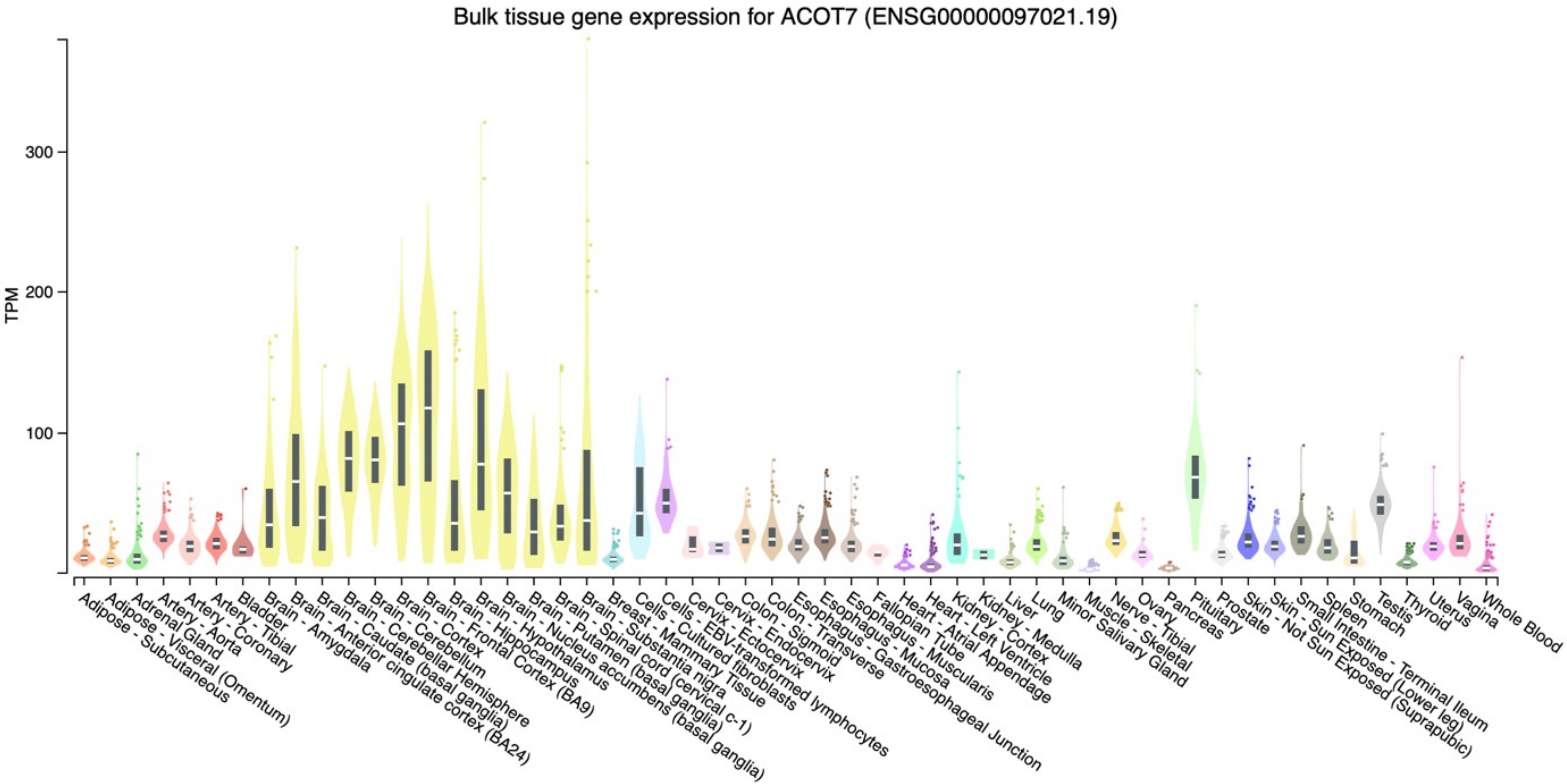

Supplementary Figure 3: ACOT7 expression profiles across tissues. Violin plots show full range, and box plots nested inside show 25% percentile (lower limit), median (middle line) and 75% percentile (higher limit).

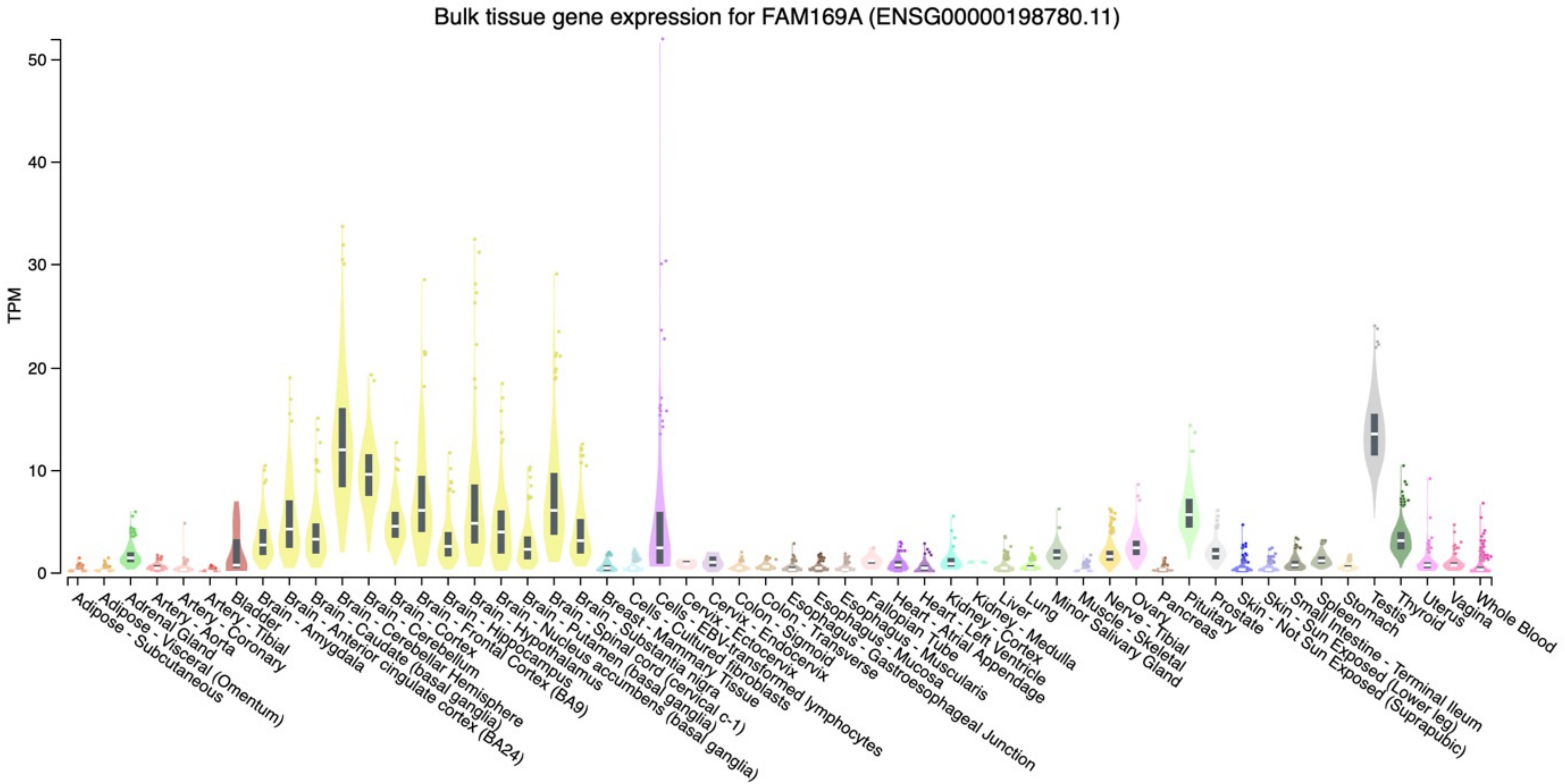

Supplementary Figure 4: FAM169A expression profiles across tissues. Violin plots show full range, and box plots nested inside show 25% percentile (lower limit), median (middle line) and 75% percentile (higher limit).

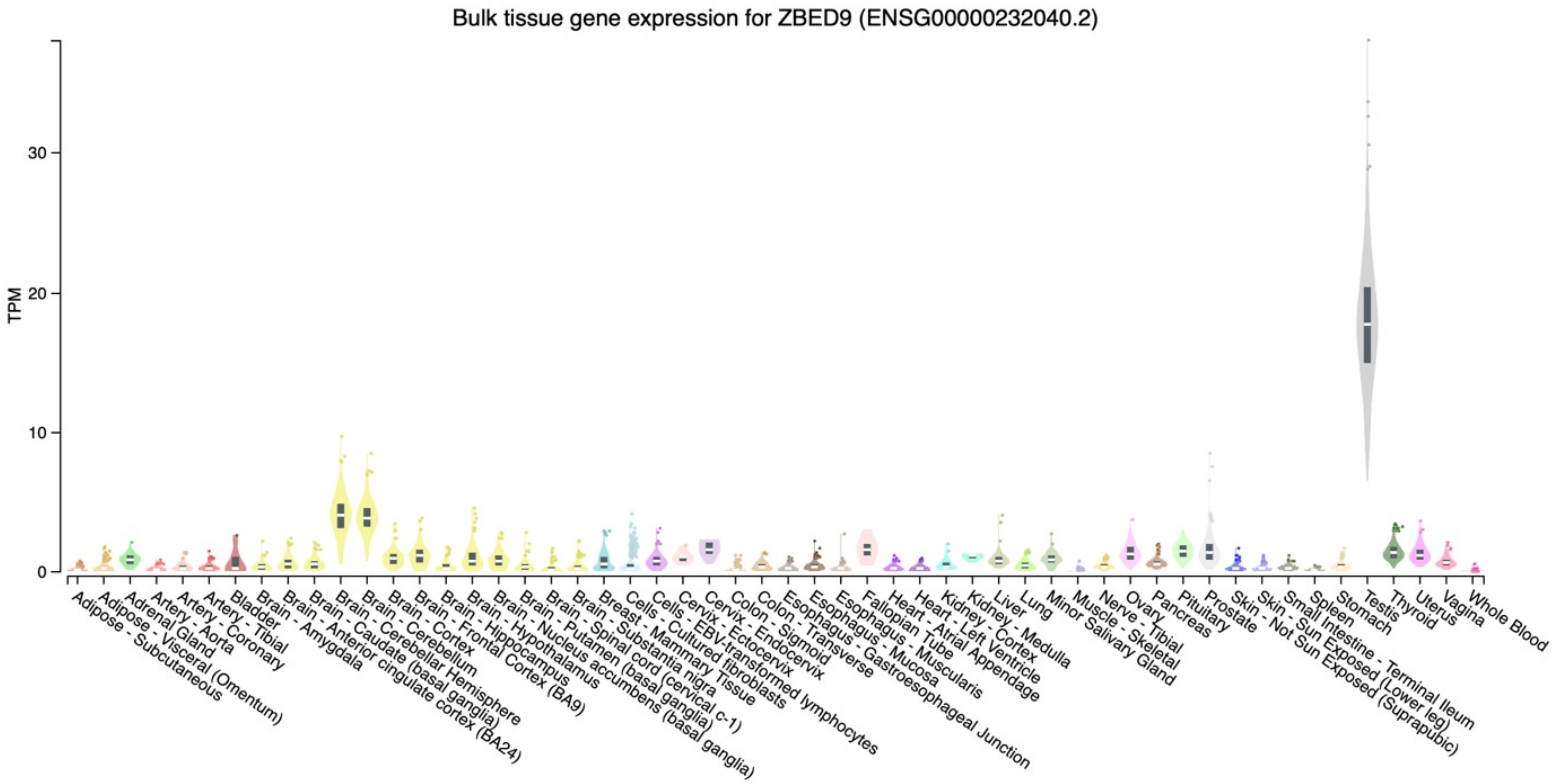

Supplementary Figure 5: ZBED9 expression profiles across tissues. Violin plots show full range, and box plots nested inside show 25% percentile (lower limit), median (middle line) and 75% percentile (higher limit).

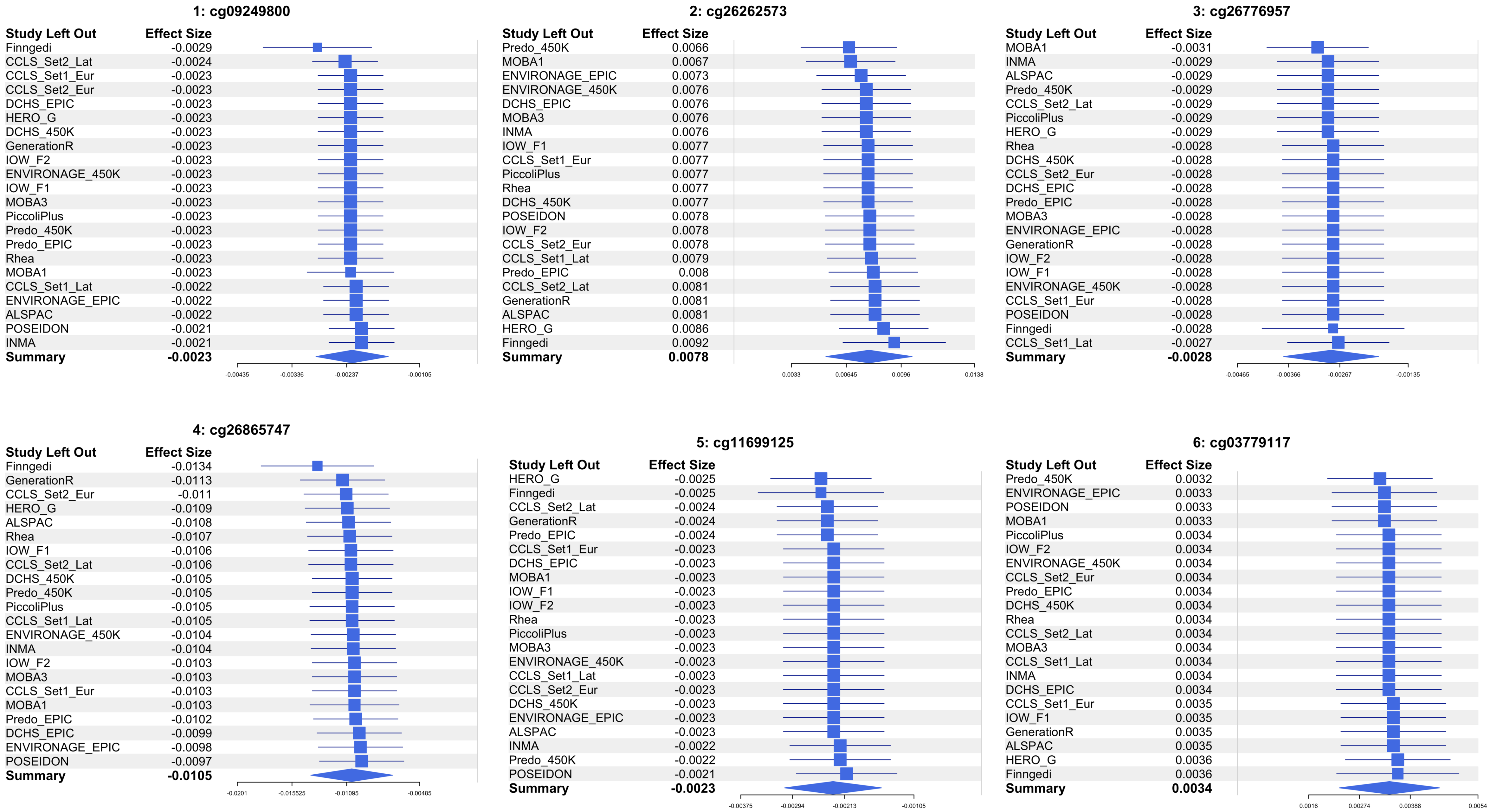

Supplementary Figure 6: Leave-One-Out analyses for top 6 CpGs from the birth-order meta-analysis results, ordered by significance level from the original model. Error bars show 95% confidence interval (CI).

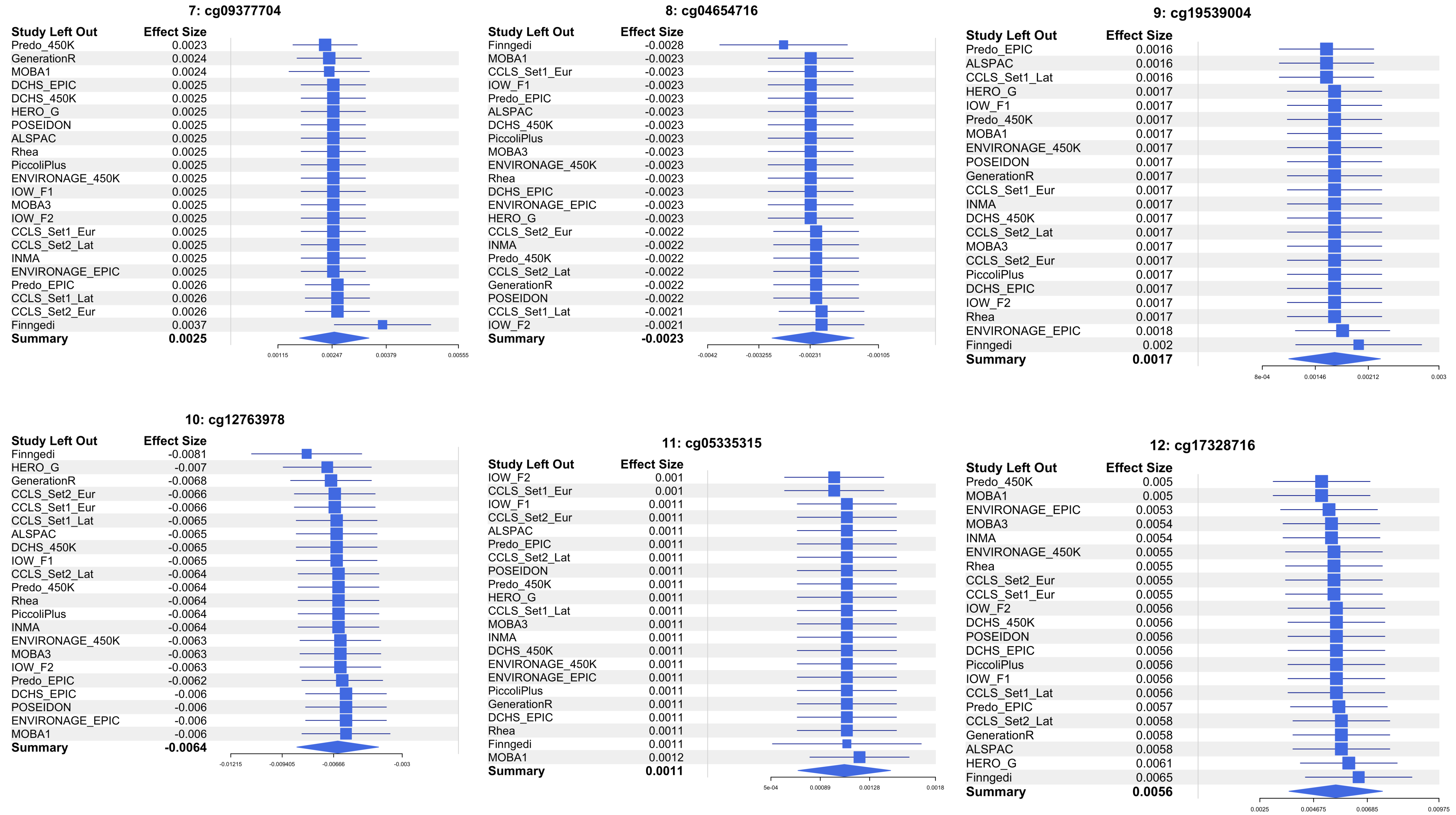

Supplementary Figure 7: Leave-One-Out analyses for top 7-12 CpGs from the birth-order meta-analysis results. Error bars show 95% CI.

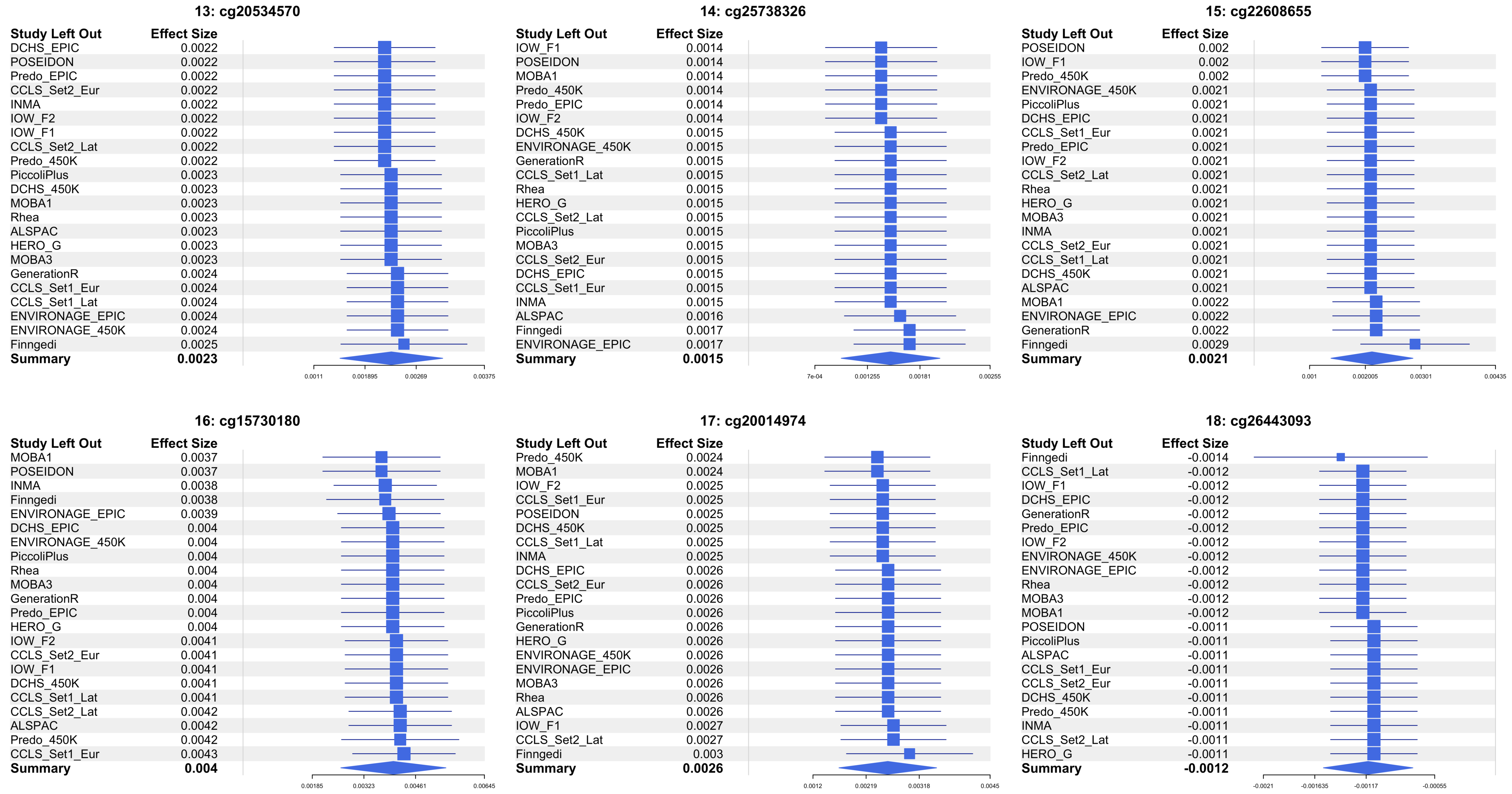

Supplementary Figure 8: Leave-One-Out analyses for top 13-18 CpGs from the birth-order meta-analysis results. Error bars show 95% CI.

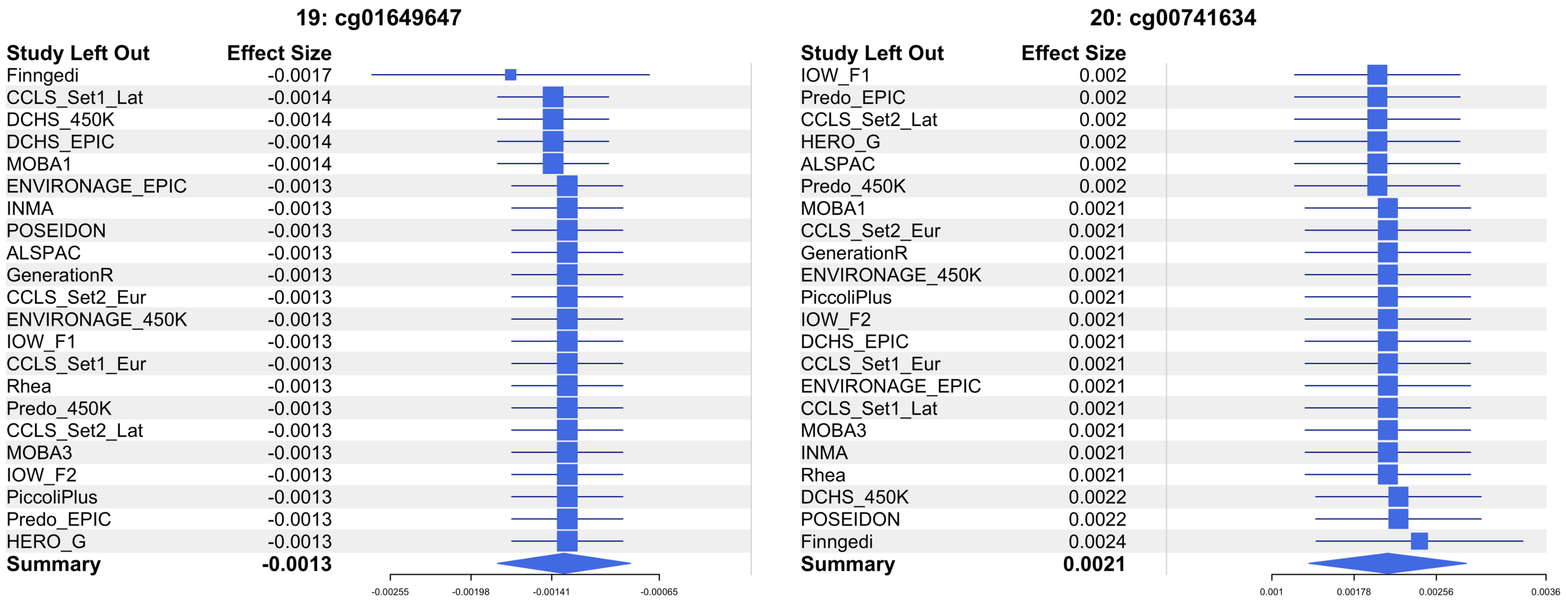

Supplementary Figure 9: Leave-One-Out analyses for top 19-20 CpGs from the birth-order meta-analysis results. Error bars show 95% CI.
