# Supplementary material for: A Pregnancy and Childhood Epigenetics Consortium (PACE) meta-analysis highlights potential relationships between birth order and neonatal blood DNA methylation
Source: Commun Biol. 2024 Jan 9;7:66. doi: 10.1038/s42003-023-05698-x (PMC10776586; doi:10.1038/s42003-023-05698-x)
Supplement: Supplementary file 3 — Description of Additional Supplementary Files [file 42003_2023_5698_MOESM3_ESM.pdf]

Description of Additional Supplementary Files

File name: Supplementary Data 1
Description: Significant DMPs associated with birth order from meta-analysis

File name: Supplementary Data 2
Description: CpGs at chromosome 7 peak overlapping LOC650226 and ZNF727

File name: Supplementary Data 3
Description: Significant DMPs associated with birth order from meta-analysis in bivariate models

File name: Supplementary Data 4
Description: Significant KEGG and GO pathways enriched by FDR significant birth order related CpGs

File name: Supplementary Data 5
Description: Trait enrichment of birth-order related CpGs

File name: Supplementary Data 6
Description: eQTMs in birth-order associated CpGs

File name: Supplementary Data 7
Description: Association between blood and brain methylations

File name: Supplementary Data 8
Description: Significant DMRs associated with birth order from meta-analysis

File name: Supplementary Data 9
Description: Significant functional annotations enriched by birth order related DMRs

File name: Supplementary Data 10
Description: Significant DMPs associated with birth order from meta-analysis in European and African Participants
