# Supplementary material for: A Pregnancy and Childhood Epigenetics Consortium (PACE) meta-analysis highlights potential relationships between birth order and neonatal blood DNA methylation
Source: Commun Biol. 2024 Jan 9;7:66. doi: 10.1038/s42003-023-05698-x (PMC10776586; doi:10.1038/s42003-023-05698-x)
Supplement: Supplementary file 5 — Reporting Summary [file 42003_2023_5698_MOESM5_ESM.pdf]

Corresponding author(s): Joseph L. Wiemels

Last updated by author(s): 12/01/2023

Reporting Summary

Nature Portfolio wishes to improve the reproducibility of the work that we publish. This form provides structure for consistency and transparency in reporting. For further information on Nature Portfolio policies, see our Editorial Policies and the Editorial Policy Checklist.

Statistics

For all statistical analyses, confirm that the following items are present in the figure legend, table legend, main text, or Methods section.

n/a	Confirmed	
☐	☒	The exact sample size (*n*) for each experimental group/condition, given as a discrete number and unit of measurement
☒	☐	A statement on whether measurements were taken from distinct samples or whether the same sample was measured repeatedly
☐	☒	The statistical test(s) used AND whether they are one- or two-sided<br>*Only common tests should be described solely by name; describe more complex techniques in the Methods section.*
☐	☒	A description of all covariates tested
☐	☒	A description of any assumptions or corrections, such as tests of normality and adjustment for multiple comparisons
☐	☒	A full description of the statistical parameters including central tendency (e.g. means) or other basic estimates (e.g. regression coefficient) AND variation (e.g. standard deviation) or associated estimates of uncertainty (e.g. confidence intervals)
☐	☒	For null hypothesis testing, the test statistic (e.g. *F*, *t*, *r*) with confidence intervals, effect sizes, degrees of freedom and *P* value noted<br>*Give P values as exact values whenever suitable.*
☒	☐	For Bayesian analysis, information on the choice of priors and Markov chain Monte Carlo settings
☒	☐	For hierarchical and complex designs, identification of the appropriate level for tests and full reporting of outcomes
☐	☒	Estimates of effect sizes (e.g. Cohen's *d*, Pearson's *r*), indicating how they were calculated

Our web collection on statistics for biologists contains articles on many of the points above.

Software and code

Policy information about availability of computer code

Data collection	No software was used in data collection.
Data analysis	Extraction of blood samples, isolation of genomic DNA, and DNA methylation array measurements were done separately by each cohort. See Supplement Methods for a detailed description for each cohort. The Illumina450K array was used by 14 cohorts and the EPIC array by 8 cohorts.Epigenome-wide association (EWAS) models were run in each cohort independently, with a prespecified pipeline using robust linear regression. If participants from a cohort included multiple ancestries, each ancestry was run separately. In total, there were 23 datasets, each including one ancestry from a specific cohort.Briefly, winsorized DNA methylation beta value for each CpG was modeled as the dependent variable, with birth order (coded as 1, 2, 3,...) as a discrete independent variable. Covariates included child sex (male as 0, female as 1), technical variable to address potential batch effects, cell type proportional estimates based on the Salas et al. cord blood reference panel 24, selection factor (if there was selection on a phenotype to create the original DNA methylation dataset for each individual study – for instance leukemia status (case/control) in the CCLS study), maternal age (years), gestational age (weeks), birthweight (gram), and maternal smoking status (nonsmoker as 0, smoker as 1). Note that despite the “selection factor” all children were not identified as such at birth – any conditions or diseases selected were diagnosed/developed later in childhood.

For manuscripts utilizing custom algorithms or software that are central to the research but not yet described in published literature, software must be made available to editors and reviewers. We strongly encourage code deposition in a community repository (e.g. GitHub). See the Nature Portfolio guidelines for submitting code & software for further information.

Data

Policy information about availability of data

All manuscripts must include a data availability statement. This statement should provide the following information, where applicable:

- Accession codes, unique identifiers, or web links for publicly available datasets
- A description of any restrictions on data availability
- For clinical datasets or third party data, please ensure that the statement adheres to our policy

Blood samples and raw genetic data of neonatal subjects from each cohort are governed by their respective institutions and/or government agencies, and mostly could not be shared publicly without specific approvals. For example, for data from first author cohort, California Childhood Leukemia Study (CCLS), we respectfully are unable to share raw, individual genetic data freely with other investigators. Should we be contacted by other investigators who would like to use the data; we will direct them to the California Department of Public Health Institutional Review Board to establish their own approved protocol to utilize the data, which can then be shared peer-to-peer.

Research involving human participants, their data, or biological material

Policy information about studies with human participants or human data. See also policy information about sex, gender (identity/presentation), and sexual orientation and race, ethnicity and racism.

Reporting on sex and gender	Sex was controlled for in regression model to adjust for sex-related differences in DNA methylations.
Reporting on race, ethnicity, or other socially relevant groupings	Regression models were done separately in each ethnicity group, to control for ethnicity-related differences in DNA methylations. Furthermore, ethnicity related principal components were adjusted for in regression models to address heterogeneity in each ethnicity group.
Population characteristics	A detailed description of mean and standard deviation of maternal ages from each of the 16 cohorts were listed in Table 1 of the manuscript. We also reported neonatal sex, birth weight and birth order variables in Table 1 from each of the cohort.
Recruitment	Each cohort had their own recruitment protocol as described in supplement material. For first author cohort, California Childhood Leukemia Study, CCLS involved recruitment at multiple facilities. Cases are subjects diagnosed with ALL in 16 participating hospitals in California. with low probability of self selection and bias in terms of birth order.
Ethics oversight	Each cohort acquired individual site-specific ethics approval and this overall analysis was approved by the University of Southern California Institutional Review Board in Health Science.

Note that full information on the approval of the study protocol must also be provided in the manuscript.

Field-specific reporting

Please select the one below that is the best fit for your research. If you are not sure, read the appropriate sections before making your selection.

☒ Life sciences ☐ Behavioural & social sciences ☐ Ecological, evolutionary & environmental sciences

For a reference copy of the document with all sections, see nature.com/documents/nr-reporting-summary-flat.pdf

Life sciences study design

All studies must disclose on these points even when the disclosure is negative.

Sample size	N= 8,164 participants were included from our study from 16 cohorts in 12 countries (Germany, South Africa, Belgium, United Kingdom, Norway, Italy, Greece, Finland, Gambia, Spain, Netherlands, United States of America). Given the commonly underpowered EWAS studies, as well as the large number of covariates in the model, we included all the available samples in our study.
Data exclusions	Only singletons, and whose older siblings are also singletons, were included in this project, if such information is available. If multiple participants within a sample set were from the same family, only one of them was randomly included in this study. Miscarriages and abortions were not counted as delivery events.
Replication	A separate replication study was not possible due to constraint of sample size, however, the meta-analysis nature of this study serves as cross-study validation, by showing consistency between multiple datasets of each CpG.
Randomization	Randomization was used in DNA methylation data preprocessing by each cohort, as described in supplement methods. For example, for CCLS cohort, each beadBhip has random samples, and included subjects of randomized birth orders.
Blinding	On cohort-level analyses, variable collection was independent of data analyses. Meta-analysis and shadow meta-analysis were done independently by two institutions with consistent results.

Reporting for specific materials, systems and methods

We require information from authors about some types of materials, experimental systems and methods used in many studies. Here, indicate whether each material, system or method listed is relevant to your study. If you are not sure if a list item applies to your research, read the appropriate section before selecting a response.

Materials & experimental systems

n/a	Involved in the study
☒	☐ Antibodies
☒	☐ Eukaryotic cell lines
☒	☐ Palaeontology and archaeology
☒	☐ Animals and other organisms
☒	☐ Clinical data
☒	☐ Dual use research of concern
☒	☐ Plants

Methods

n/a	Involved in the study
☒	☐ ChIP-seq
☒	☐ Flow cytometry
☒	☐ MRI-based neuroimaging

Plants

Seed stocks	NA
Novel plant genotypes	NA
Authentication	NA
